# alpha-Synuclein: a Modulator During Inflammatory CNS Demyelination

**DOI:** 10.1007/s12031-020-01498-8

**Published:** 2020-03-23

**Authors:** Kristina Kuhbandner, Alana Hoffmann, María Nazareth González Alvarado, Lisa Seyler, Tobias Bäuerle, Jürgen Winkler, Ralf A. Linker

**Affiliations:** 1 Department of Molecular Neurology, University Hospital Erlangen, Friedrich-Alexander-University Erlangen-Nürnberg, Schwabachanlage 6, 91054 Erlangen, Germany; 2 Department of Neurology, University Hospital Erlangen, Friedrich-Alexander-University Erlangen-Nürnberg, Erlangen, Germany; 3 Department of Neurology, University of Regensburg, Regensburg, Germany; 4 Institute of Radiology, Preclinical Imaging Platform Erlangen (PIPE), University Hospital Erlangen, Friedrich-Alexander-University Erlangen-Nürnberg, Erlangen, Germany

**Keywords:** Neuroinflammation, Demyelination, alpha-Synuclein, Experimental autoimmune encephalomyelitis, Cuprizone

## Abstract

Neuroinflammation and demyelination are hallmarks of several neurological disorders such as multiple sclerosis and multiple system atrophy. To better understand the underlying mechanisms of de- and regeneration in respective diseases, it is critical to identify factors modulating these processes. One candidate factor is alpha-Synuclein (aSyn), which is known to be involved in the pathology of various neurodegenerative diseases. Recently, we have shown that aSyn is involved in the modulation of peripheral immune responses during acute neuroinflammatory processes. In the present study, the effect of aSyn deficiency on de- and regenerative events in the CNS was analyzed by using two different demyelinating animal models: chronic MOG_35–55_-induced experimental autoimmune encephalomyelitis (EAE) and the cuprizone model. Histopathological analysis of spinal cord cross sections 8 weeks after EAE induction revealed a significant reduction of CNS inflammation accompanied by decreased myelin loss during late-stage inflammatory demyelination in aSyn-deficient mice. In contrast, after cuprizone-induced demyelination or remyelination following withdrawal of cuprizone, myelination and neuroinflammatory patterns were not affected by aSyn deficiency. These data provide further evidence for aSyn as regulator of peripheral immune responses under neuroinflammatory conditions, thereby also modulating degenerative events in late-stage demyelinating disease.

## Introduction

Demyelination in combination with neuroinflammatory responses is a common feature of several neurological disorders including multiple system atrophy (MSA) and multiple sclerosis (MS) (Compston and Coles 2008; Fanciulli and Wenning 2015). Physiologically, compensating repair mechanisms exist which sustain myelin regeneration. While in some MS patients these processes frequently occur, others only show sparse remyelination (Patrikios et al. 2006). The reasons for this regeneration failure still remain unclear. Up to date, no therapeutic options exist to cure these diseases or to halt disease progression. For the development of such treatment strategies, it is essential to identify factors which are involved in de- and remyelinating processes as well as oligodendrocyte cell damage and death. In rodent models, it has been shown that remyelination critically depends on the presence of oligodendrocyte precursor cells (OPCs) (Sim et al. 2002). In the central nervous system (CNS), OPCs are recruited to demyelinated areas and give rise to myelinating oligodendrocytes upon activation (Franklin and ffrench-Constant 2017). Current concepts to explain the failure of remyelination include insufficient recruitment of OPCs to the demyelinated region or the inhibition of maturation into oligodendrocytes (Sim et al. 2002; Kuhlmann et al. 2008). So far, various extrinsic and intrinsic factors modulating oligodendrocyte maturation have been described (French et al. 2009; Hughes et al. 2013; He et al. 2016). Recent studies using in vitro and in vivo MSA models suggest alpha-Synuclein (aSyn) as a candidate factor inhibiting OPC maturation (Ettle et al. 2014; May et al. 2014). aSyn is expressed in axons, neuritic processes, and presynaptic terminals of neurons, being important for vesicle transport. In addition, aSyn protein levels have also been detected in glia and hematopoietic cells (Maroteaux et al. 1988; Barbour et al. 2008). Pathological aSyn aggregations are associated with neurodegenerative disorders, in particular Parkinson’s disease, dementia with Lewy bodies, and MSA, also summarized as synucleinopathies (Spillantini et al. 1998). In the latter, aSyn accumulates in glial cytoplasmic inclusions (GCI) in oligodendrocytes being considered pathological hallmark and associated with widespread demyelination (Papp et al. 1989). Additionally, degenerative features are often accompanied by distinct neuroinflammatory processes in these synucleinopathies (Lim et al. 2016; Hoffmann et al. 2019). Interestingly, aSyn immunoreactivity was also reported in glial cells in MS lesions and in spinal cord lesions of mice induced with experimental autoimmune encephalomyelitis (EAE), a common animal model of MS (Papadopoulos et al. 2006; Lu et al. 2009). Recently, we identified aSyn as regulator of peripheral immune responses in neuroinflammation (Ettle et al. 2016b). Considering these findings, we studied the role of aSyn during late-stage neuroinflammatory demyelination and in the context of toxin-induced de- as well as remyelination. For this purpose, demyelination was induced in aSyn-deficient mice (aSyn^−/−^) and littermate control animals with endogenous aSyn expression (aSyn^+/+^) by using two different demyelination models. More precisely, autoimmune-mediated late-stage demyelination was examined with the help of myelin oligodendrocyte glycoprotein (MOG)_35–55_–EAE and toxin-induced demyelination was studied in the cuprizone model. Furthermore, regenerative events were assessed at two different time points after stopping cuprizone administration.

## Material and Methods

### Animal Experiments

aSyn^−/−^ mice were maintained on a C57BL/6N background for more than ten generations (Abeliovich et al. 2000). aSyn^+/+^ littermates with endogenous aSyn expression served as controls. All mice were kept under standard animal housing conditions with a 12-h day/night cycle and free access to food and water. All applicable international, national, and/or institutional guidelines for the care and use of animals were followed. Animal experiments were performed in accordance with the German laws of animal protection and were approved by the local ethics committee (Government of Unterfranken, Bavaria, Germany, ref. # 55.2-2532-2-395 and # 55.2-2532-2-450).

### Experimental Autoimmune Encephalomyelitis

For active EAE induction, 10–14-week-old aSyn^+/+^ and aSyn^−/−^ mice received 200 μg MOG_35–55_ and 200 μg complete Freund’s adjuvant (CFA) subcutaneously. Pertussis toxin (200 ng) was applied intraperitoneally at the day of immunization and 48 h later. Mice were daily weighed and scored for clinical signs using a 10-point scale as described previously (Linker et al. 2002).

### Cuprizone Model

Toxic demyelination was induced by feeding 8–10-week-old aSyn^+/+^ and aSyn^−/−^ mice a diet containing 0.2% cuprizone mixed into ground standard rodent chow for 5 weeks. During this period, mice were closely monitored for weight loss as well as abnormalities and trained for Rotarod analysis. After 5 weeks, motor coordination skills were assessed by an accelerating Rotarod test. To initialize remyelination, after 5 weeks, cuprizone was removed from the diet and mice received ground standard rodent chow for 3 or 7 days, respectively. At the end of de- and remyelination experiments, mice were sacrificed to dissect the brain for histological analysis. C57BL/6N wildtype mice fed with normal chow served as naïve controls and are termed “naïve wt.”

### Rotarod Test

Motor performance of mice treated with cuprizone was evaluated by performing an accelerating Rotarod test. The Rotarod consisted of a 30-mm diameter rod rotating about its long axis. Over the course of 180 s, the Rotarod accelerated from 4 to 40 rounds per minute (rpm). The latency of each mouse to fall was recorded in two consecutive trials with a 30-min resting phase in the home cage between the trials. One week prior to the accelerating test, mice were trained on the rod rotating with constant speed at 12 rpm for 120 s.

### In Vivo Magnetic Resonance Imaging

In vivo magnetic resonance imaging (MRI) was performed with aSyn^+/+^ and aSyn^−/−^ mice before and after cuprizone administration. In inhalation anesthesia (1.5% isoflurane), heads of mice were fixed in a mouse brain surface array coil and scanned on a preclinical ultra-high field MRI system (7 Tesla ClinScan 70/30, Bruker). During the entire imaging procedure, respiration of mice was monitored and kept constant, while the body temperature was stabilized with a heating circulator bath. The imaging protocol consisted of a 3D T2-weighted turbo spin echo sequence (repetition time (TR), 3690 s; echo time (TE), 40 s; voxel size, 0.082 × 0.082 × 0.5 mm; acquisition time, 4 min 37 s; slice thickness, 0.5 mm).

Sequences were analyzed with the help of the medical imaging interaction toolkit (MITK). T2-weighted signal intensities were assessed in aSyn^+/+^ and aSyn^−/−^ mice before and after cuprizone diet. The boundaries of the region of interest (ROI) within white matter regions of the corpus callosum were manually defined (Wu et al. 2018). To eliminate confounding signals arising from slice-dependent signal variation during MRI acquisition, the signal intensity of the ventricle within the same slice was used for normalization. To calculate T2-weighted signal intensity ratios, signal intensities were determined in the ROI and the ventricle of each animal in the same layer.

### (Immuno-)Histochemistry

Following perfusion with 4% paraformaldehyde (PFA), spinal cords or brains were removed and post-fixed in 4% PFA for 2–3 h. After embedding in paraffin, 4 μm thin sections were prepared by using a microtome. For immunohistochemistry, anti-CD3 (MCA1477, Biorad) and anti-Mac-3 (M3/84, BD Pharmingen) antibodies were used to detect T cells and mononuclear phagocytes, respectively. Glial cells were stained with anti-Olig2 (Millipore), anti-NogoA (Millipore), anti-GFAP (Dako), and anti-Iba1 (20A12.1, Millipore) antibodies, respectively. Anti-MOG (8-18C5, Millipore), anti-MBP (12, Biorad), and anti-2′,3′-Cyclic-nucleotide 3′-phosphodiesterase (CNPase) (SMI91R, BioLegend) antibodies were used as myelin markers. For chromogenic immunodetection, Vectastain Elite Avidin-Biotin-Complex Kit (Vector Laboratories) was used according to the manufacturer’s instructions. Diaminobenzidine (DAB) was employed to visualize the conjugated peroxidase.

Luxol fast blue-periodic acid Schiff (LFB-PAS) staining was performed to assess the extent of demyelination. Therefore, deparaffinized slides were incubated overnight in 0.1% Luxol fast blue (LFB) solution at 56 °C. Next day, sections were rinsed in 96% ethanol and distilled water*.* For differentiation, sections were incubated in 1% lithium carbonate solution for a few seconds, rinsed shortly in 70% ethanol, and washed in distilled water. PAS staining was performed by oxidizing the slides in 0.8% periodic acid solution for 10 min*.* Afterwards, sections were stained in Schiff reagent for 20 min and washed in sulfite solution, followed by a 10-min washing step with running tap water. Finally, sections were dehydrated and mounted.

For analysis of axonal integrity, Bielschowsky silver impregnation was employed. Rehydrated sections were transferred into a 20% AgNO_3_ solution and incubated for 15 min at 37 °C. Then the solution was washed off with distilled water and 25% NH_4_OH solution was added to the AgNO_3_ solution. Slides were incubated in this solution for 10 min in the dark at 37 °C and washed in 0.1% NH_4_OH. After adding developer stock solution to the AgOH solution, sections were stained for 4 min until axons turned black. Slides were rinsed in 0.1% NH_4_OH and distilled water and fixed in 5% Na_2_S_2_O_3_ for 3 min. Finally, sections were washed in distilled water*,* dehydrated, and mounted.

### Histological Evaluation

For histological evaluation of EAE experiments, spinal cord cross sections were analyzed blinded using a BX-51 light microscope (Olympus). For each staining, at least five lesions within each spinal cord segment (cervical, thoracic, and lumbar) were inspected. T cells, mononuclear phagocytes, astrocytes, and oligodendrocytes were counted at × 200 magnification within the margins of a 1/16 mm^2^ grid in individual lesions and counts were normalized to cells/mm^2^. Analysis of demyelinated areas in the white matter of LFB and anti-CNPase-stained sections was performed semi-automatically with the help of CellSens or CellP software (Olympus). Axonal density was quantified in silver impregnated sections by counting on a 100 μm diameter grid at × 500 magnification.

To analyze brain sections in cuprizone experiments, sections between bregma − 1.15 and bregma − 2.15 according to the Mouse Brain atlas by Franklin and Paxinos were examined (Franklin and Paxinos 2013). The level of demyelination in the corpus callosum was assessed by determination of myelin scores by two independent blinded observers as follows: 0: complete demyelination, 1: 1/3 of corpus callosum myelinated, 2: 2/3 of corpus callosum myelinated, 3: complete myelination (Lindner et al. 2008).

Number of cells was determined in three adjacent regions in the median corpus callosum of each animal using a morphometric grid. Immunopositive cells with identified nucleus (counterstaining with hematoxylin) were counted at × 200 magnification and counts were normalized to cells/mm^2^.

### Statistical Analysis

Statistical testing was performed using Graph Pad Prism. All ex vivo data were analyzed by one- or two-way ANOVA followed by Tukey’s posttest or unpaired *t* test. EAE data were analyzed either by Mann-Whitney *U* test or logrank test (for disease incidence analysis). For statistical analysis of MRI data, the two-way ANOVA test for repeated measurements was used. Data are presented as mean ± SEM; *p < 0.05, **p < 0.01, or ***p < 0.001 were considered to be statistically significant.

## Results

### aSyn Deficiency Ameliorates EAE Severity

**Fig. 1:**
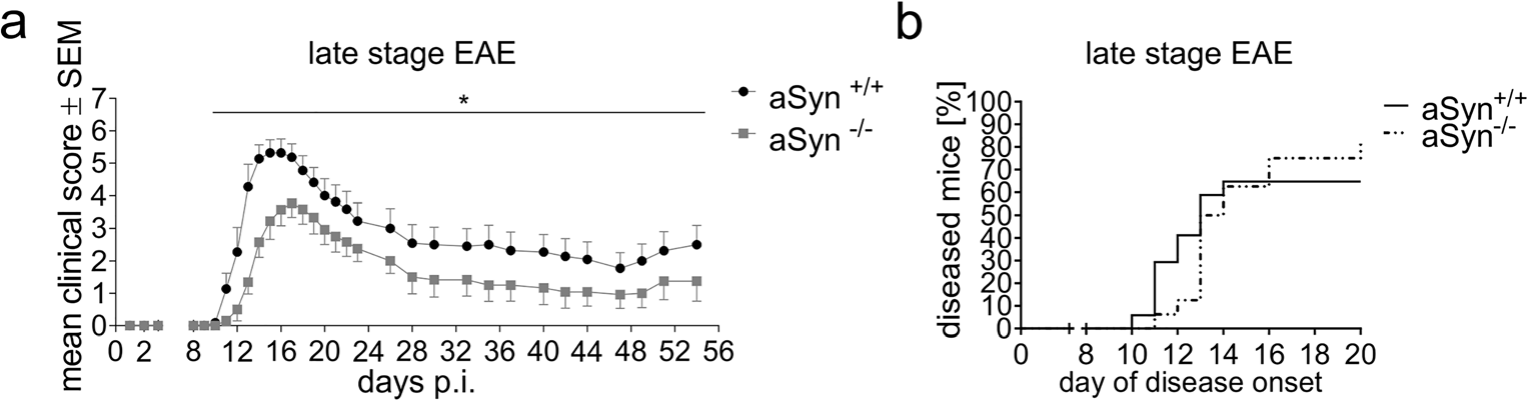
aSyn deficiency attenuates disease severity in late-stage EAE. **a** Clinical course of MOG_35–55_-EAE in aSyn^+/+^ (black dots) and aSyn^−/−^ (gray squares) mice (*n* = 11–13 per group). **b** Kaplan-Meier plot depicting EAE incidence in aSyn^+/+^ (solid line) and aSyn^−/−^ (dashed line) mice (*n* = 16–17 per group). p.i. post immunization. *p < 0.05

First, we used the EAE model to study the effect of aSyn deficiency during late-stage inflammatory demyelination. Therefore, aSyn^+/+^ and aSyn^−/−^ mice were immunized with MOG_35–55_ peptide and monitored for clinical signs over a period of 8 weeks. Compared with littermate controls, aSyn-deficient mice displayed an ameliorated disease course (Fig. 1a). At the end of the observation period, they showed a better outcome by one score point in comparison with aSyn^+/+^ mice. More precisely, they suffered from tail paralysis, while the control group still exhibited signs of gait ataxia and mild paralysis of hind limbs. There were no differences in the overall disease incidence or mortality rate between both groups (Fig. 1b).

### Reduced Demyelination, CNS Inflammation, and Axonal Loss in aSyn-Deficient Mice During Late-Stage EAE

**Fig. 2:**
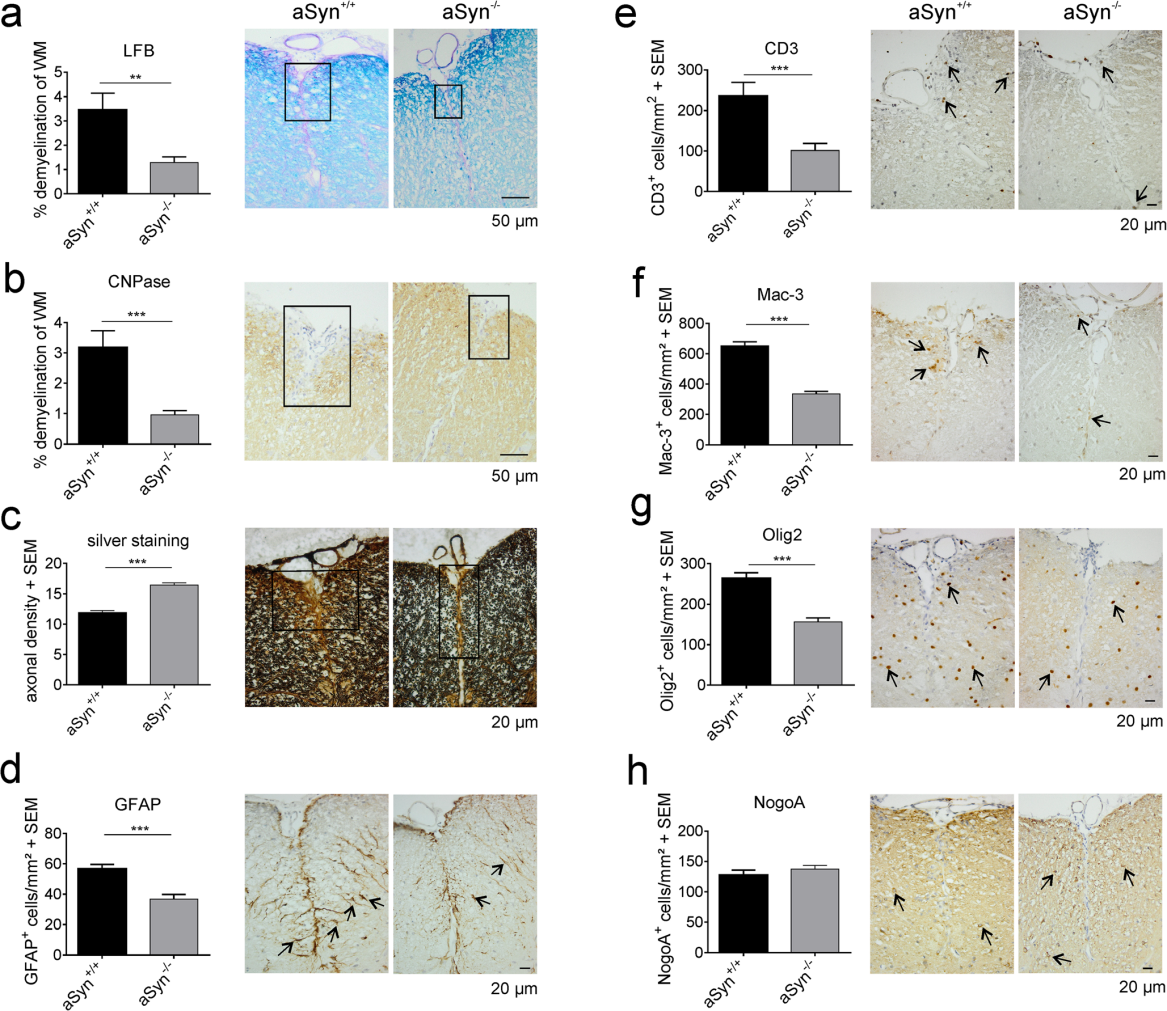
aSyn-deficient mice exhibit less severe demyelination, axonal loss and inflammation in CNS lesions. (Immuno-)Histochemical analysis of spinal cord cross sections of aSyn^+/+^ (black bars) and aSyn^−/−^ (gray bars) mice 8 weeks after immunization with MOG_35–55_ peptide. Average clinical scores at the time point of analysis were 1.38 for aSyn^−/−^ mice and 2.5 for aSyn^+/+^ mice. Percentage of demyelinated area in the white matter area determined by **a** LFB-PAS staining or **b** staining with anti-CNPase. **c** Bar graph depicting axonal densities (number of axons counted on a 100 μm grid) determined by Bielschowsky silver staining. Numbers of **d** GFAP^+^ astrocytes, infiltrating **e** CD3^+^ and **f** Mac-3^+^ cells, **g** Olig2^+^ and **h** NogoA^+^ cells were assessed by immunohistochemical staining. Representative pictures are shown for each staining (*n* = 5–6 per group). **p < 0.01, ***p < 0.001

In the EAE model, disease course and severity of symptoms are determined by the extent of CNS inflammation and spinal cord damage is mainly apparent in a form of demyelination and axonal loss. Therefore, we histologically analyzed spinal cords of aSyn^+/+^ and aSyn^−/−^ mice for these parameters 8 weeks after induction of EAE. Average clinical scores at this time point were 1.38 for aSyn^−/−^ mice and 2.5 for aSyn^+/+^ mice, respectively. Matching the disease course, immunohistochemical staining for CNPase as well as LFB-PAS staining revealed a more than 2.5-fold increase in white matter demyelination in aSyn^+/+^ mice (Fig. 2a, b). Furthermore, axonal densities determined by Bielschowsky silver staining were reduced by 30% in lesions of aSyn^+/+^ mice compared with aSyn^−/−^ mice (Fig. 2c). Additionally, we detected a marked increase in GFAP^+^ processes and significantly higher numbers of GFAP^+^ cell bodies within and in direct vicinity of spinal cord lesions in these mice indicative for astrocytic activation (Fig. 2d). Further examination of neuroinflammatory processes revealed a pronounced inflammatory response in aSyn^+/+^ mice. Overall, fewer lesions were present in aSyn^−/−^ mice containing a lower number of invading immune cells. More specifically, CD3^+^ T cells and Mac-3^+^ mononuclear phagocytes infiltrating the CNS were reduced by 50% compared with aSyn^+/+^ animals (Fig. 2e, f). To closer examine the effect of aSyn deficiency on oligodendroglia, numbers of NogoA^+^ and Olig2^+^ cells were determined in demyelinated areas. While the number of NogoA^+^ mature oligodendrocytes did not differ between both groups, significantly more cells positive for Olig2^+^, a relatively broad oligodendrocytic marker, were detected in lesions of aSyn^+/+^ mice (Fig. 2g, h).

### aSyn Deficiency Does Not Affect Myelination Pattern After Cuprizone-Induced Demyelination

**Fig. 3:**
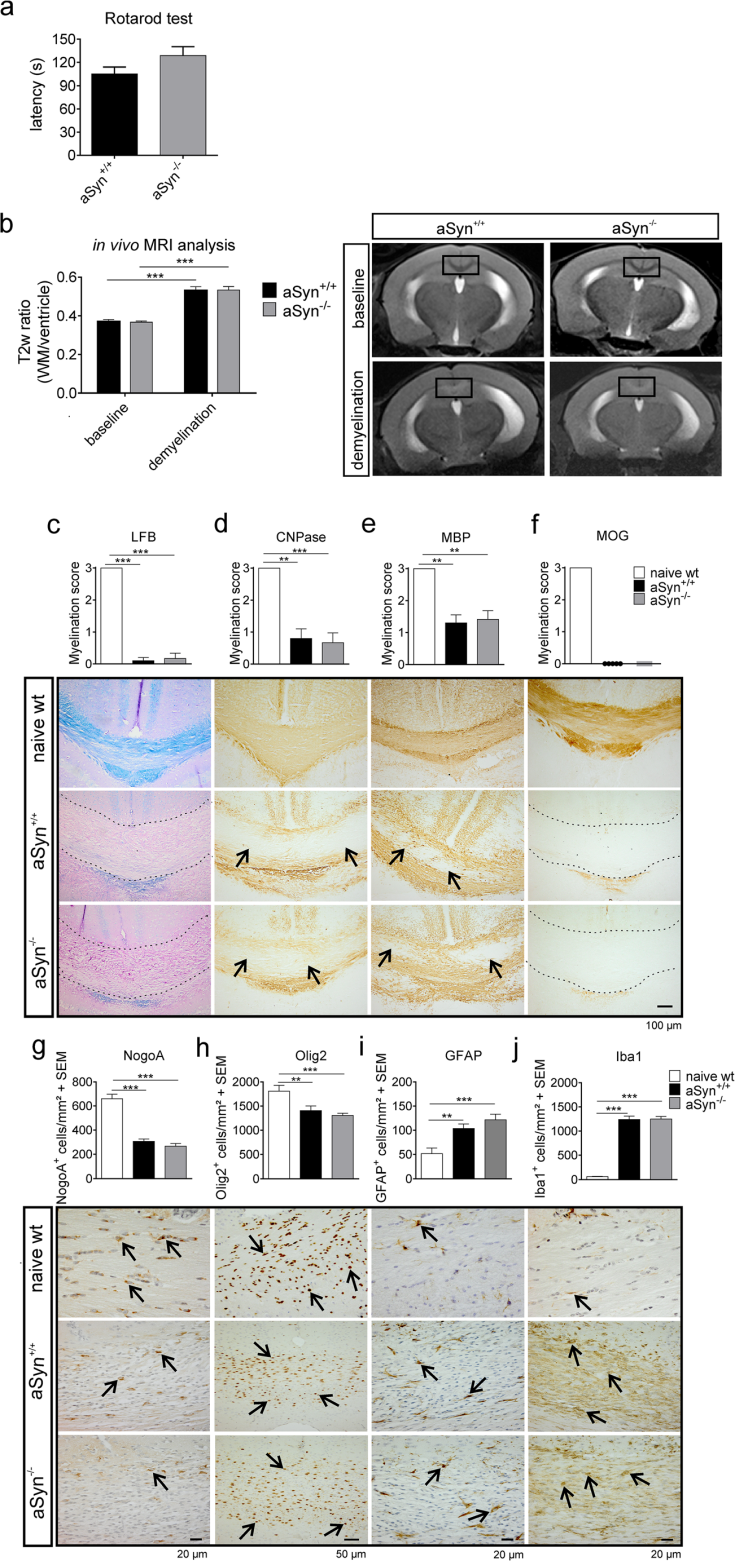
aSyn deficiency does not affect acute demyelination after 5 weeks of cuprizone treatment. aSyn^+/+^ (black bars) and aSyn^−/−^ (gray bars) mice received cuprizone diet for 5 weeks. **a** Motor coordination was tested by an accelerating Rotarod test (*n* = 5–6 per group). **b** T2-weighted (T2w) signal intensity ratios (white matter in corpus callosum and liquor) were determined by in vivo MRI in aSyn^+/+^ and aSyn^−/−^ mice before (baseline) and after 5 weeks of cuprizone diet (demyelination; *n* = 5–6 per group; WM, white matter). Representative MRI images are presented with parts of the corpus callosum used for analysis shown in black frames. Demyelination patterns in the corpus callosum were assessed by (immuno-) histochemical staining with **c** LFB, **d** anti-CNPase, **e** anti-MBP, or **f** anti-MOG antibody. Numbers of CNS cells in the corpus callosum were determined by staining with **g**) anti-NogoA, **h**) anti-Olig2 **i** anti-GFAP, or **j** anti-Iba1 antibody staining (naïve wt: *n* = 3; aSyn = 4–6 per group). **p < 0.01, ***p < 0.001

Next to the EAE model, the cuprizone model was employed to investigate the impact of aSyn deficiency on acute demyelination and remyelinating processes in the absence of peripheral immune responses. To evoke acute demyelination in the brain of aSyn^+/+^ and aSyn^−/−^ mice, age-matched animals were fed a diet containing 0.2% cuprizone for 5 weeks. Motor coordination skills of mice were assessed after cuprizone treatment by an accelerating Rotarod test. Both groups showed equal performances regarding latency to stay on the accelerating rod (Fig. 3a). In vivo MRI was used to detect myelin deficits in the corpus callosum following cuprizone treatment. After 5 weeks of cuprizone diet, gray-white matter contrast was conspicuously reduced. Quantification of T2-weighted signal intensity ratios of white matter and liquor revealed a 70% increase in animals receiving cuprizone diet, indicating a considerable reduction in myelin content in the corpus callosum. However, this method did not detect any differences in demyelination patterns between aSyn^+/+^ and aSyn^−/−^ mice following cuprizone exposure (Fig. 3b). To validate these findings, a comprehensive histological analysis of brain sections was performed. In line with results obtained from the MRI measurement, immunohistochemical staining for different myelin markers, such as MBP, CNPase, and MOG, as well as LFB-PAS staining demonstrated that 5 weeks of cuprizone treatment resulted in comparable reduction of myelin staining in both, aSyn^+/+^ and aSyn^−/−^ mice, in the central part of the corpus callosum compared with naïve wt mice (Fig. 3c–f). Additionally, the impact on CNS cells was studied. Cuprizone treatment strongly reduced the number of mature NogoA^+^ oligodendrocytes compared with naïve mice (Fig. 3g). However, no differences were observed in the numbers of mature NogoA^+^ oligodendrocytes or Olig2^+^ cells in the corpus callosum between aSyn^+/+^ and aSyn^−/−^ mice (Fig. 3g, h). Following cuprizone administration, neuroinflammatory processes including activation of astrocytes and microglia are induced (Hiremath et al. 1998). Indeed, numbers of Iba1^+^ microglia and GFAP^+^ astrocytes were markedly increased by 14-fold and twofold, respectively, in the corpus callosum of aSyn^+/+^ and aSyn^−/−^ mice treated with cuprizone for 5 weeks compared with untreated controls. However, in aSyn^+/+^ and aSyn^−/−^ mice, numbers of respective cells were nearly equal (Fig. 3i, j).

### aSyn Deficiency Has No Impact on Early Remyelination in the Cuprizone Model

**Fig. 4:**
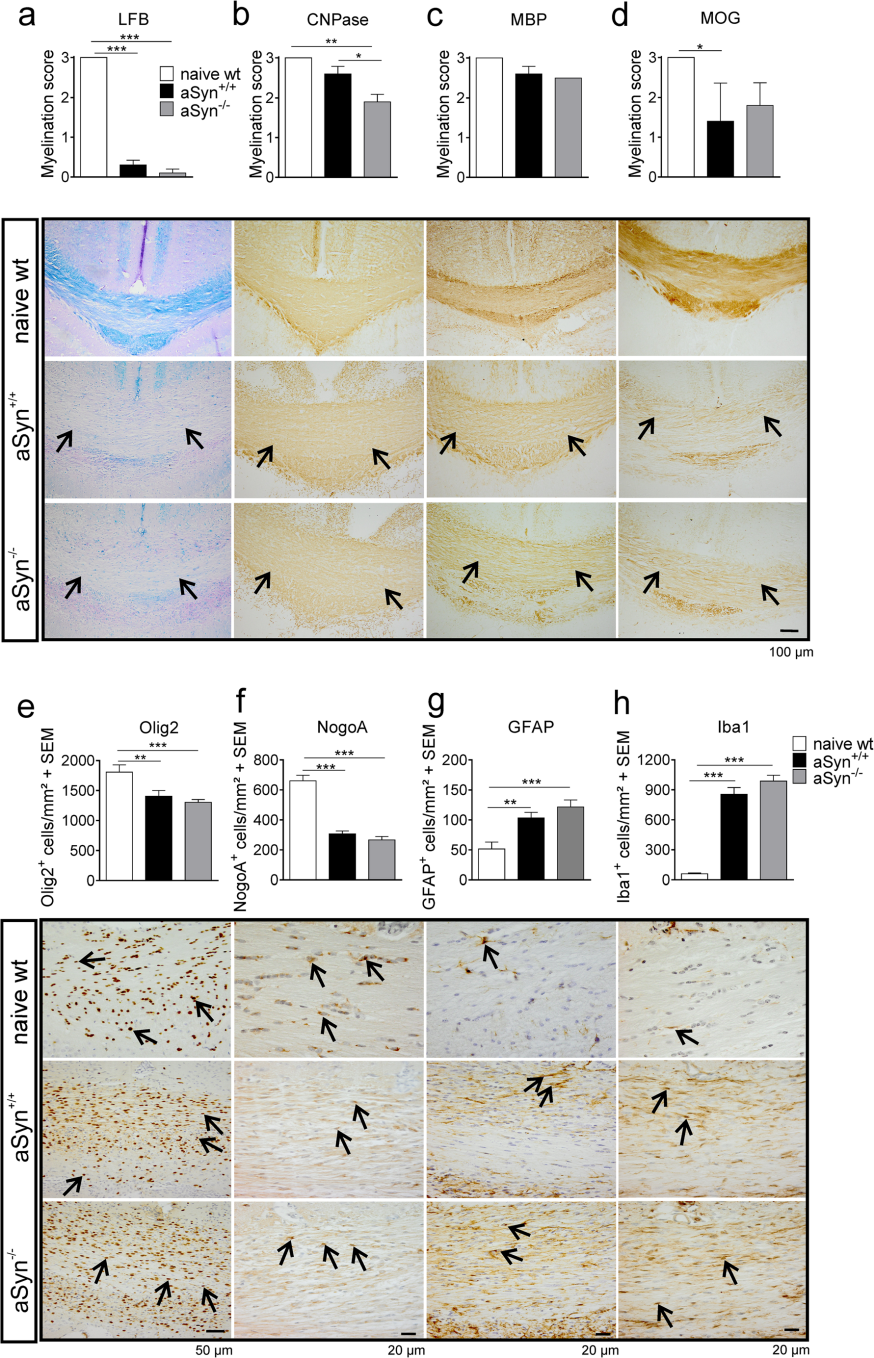
Early remyelinating processes are similar in aSyn^−/−^ and aSyn^+/+^ mice. Three days after terminating the cuprizone diet, remyelination in the corpus callosum of aSyn^+/+^ (black bars) and aSyn^−/−^ (gray bars) mice was histologically assessed by (immuno-) histochemical staining with **a** LFB/PAS or antibodies against **b** CNPase, **c** MBP, or **d** MOG. Numbers of CNS cells were analyzed by staining with antibodies against **e** Olig2, **f** NogoA, **g** GFAP, or **h** Iba1 (naïve wt: *n* = 3; aSyn = 4–6 per group). **p < 0.01, ***p < 0.001

**Fig. 5:**
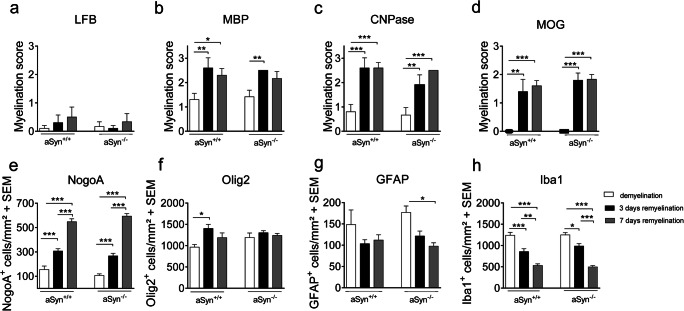
Similar de- and regenerative patterns in aSyn^+/+^ and aSyn^−/−^ mice following cuprizone treatment. Comparison of de- and remyelination patterns and CNS cell numbers in the corpus callosum of aSyn^+/+^ (black bars) and aSyn^−/−^ (gray bars) mice at different time points. Histological analysis was performed by staining with **a** LFB/PAS or antibodies against **b**) MBP, **c**) CNPase, or **d** MOG, **e**) NogoA, **f**) Olig2, **g** GFAP, or **h** Iba1. The time points investigated were demyelination (5 weeks after cuprizone treatment), 3 days after stopping the cuprizone diet (3 days remyelination), and 7 days after termination of the cuprizone treatment (7 days remyelination) (aSyn = 3–5 per group and time point). *p < 0.05, **p < 0.01, ***p < 0.001

**Fig. 6:**
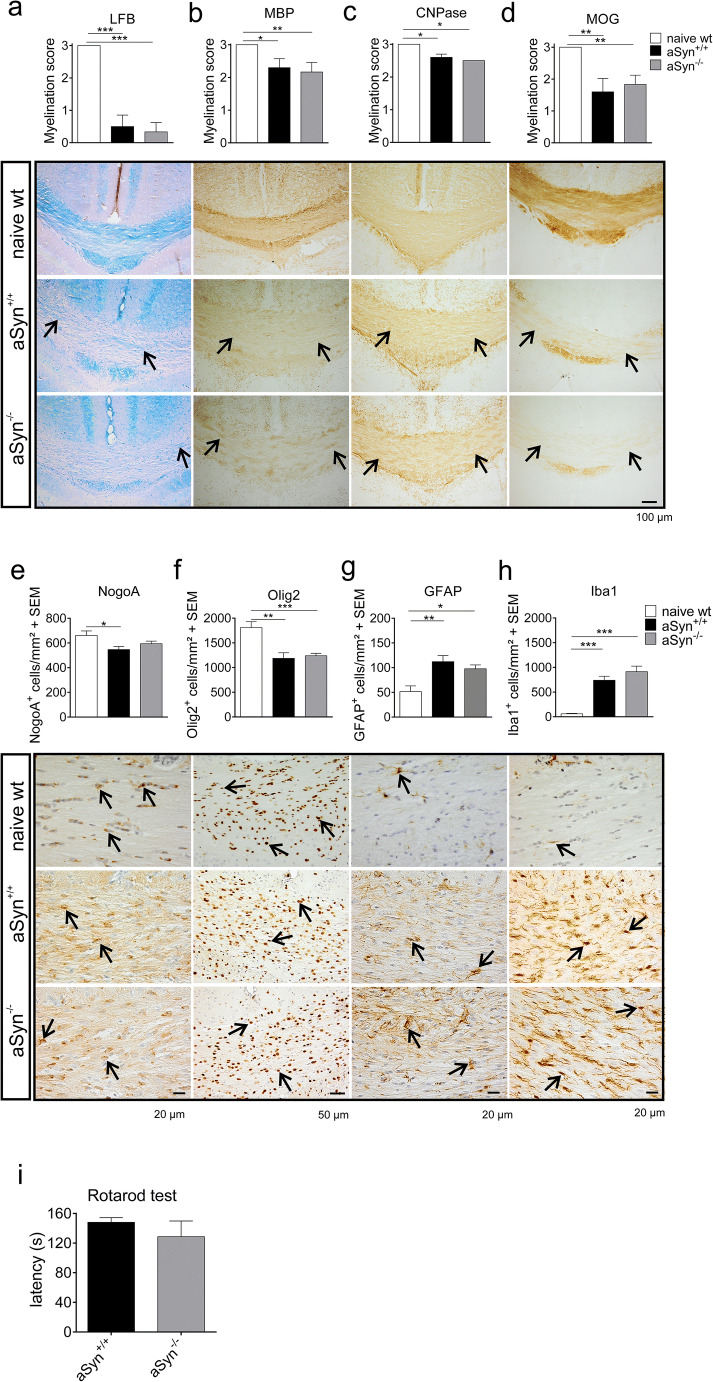
aSyn deficiency does not alter remyelination processes 1 week after terminating cuprizone administration. Seven days after stopping the cuprizone diet, remyelination in the corpus callosum of aSyn^+/+^ (black bars) and aSyn^−/−^ (gray bars) mice was histologically assessed by (immuno-) histochemical staining with **a** LFB/PAS or antibodies against **b**) MBP, **c**) CNPase, or **d** MOG. Numbers of CNS cells were analyzed by staining with antibodies against **e**) NogoA, **f**) Olig2, **g** GFAP, or **h** Iba1 (naïve wt: *n* = 3; aSyn = 3–5 per group). **i** Motor coordination skills were assessed in aSyn^+/+^ and aSyn^−/−^ animals 7 days after withdrawal of cuprizone using an accelerating Rotarod test (*n* = 5 per group). *p < 0.05, **p < 0.01, ***p < 0.001

The cuprizone model is considered a well-established tool to investigate remyelination since, only few days after administration, regenerative processes are emerging. In order to study the effect of aSyn deficiency on early remyelination, we examined aSyn^+/+^ and aSyn^−/−^ mice for their myelination pattern by histological analysis 3 days after terminating the cuprizone diet. In both groups, myelination scores assessed by histological staining for myelin markers (MOG, MBP) and LFB staining were similar in aSyn^+/+^ and aSyn^−/−^ mice (Fig. 4a–d). Compared with myelin analysis determined at the time point of full demyelination, MOG and MBP scores were significantly higher in both groups, further indicating the presence of remyelination processes (Fig. 5b, d, Appendix). Analogous to the results obtained at the time point of full demyelination, also numbers of Olig2^+^ and NogoA^+^ oligodendroglia as well as numbers of GFAP^+^ cells and Iba1^+^ microglia were similar in aSyn^+/+^ and aSyn^−/−^ mice (Fig. 4e–h). Notably, in both groups, the number of mature NogoA^+^ oligodendrocytes was increased by about 50% compared with the cell number detected in the corpus callosum after 5 weeks of cuprizone diet, suggesting recovery of the oligodendrocyte population after marked toxin-induced reduction (Fig. 5e, Appendix). Additionally, regenerative processes were assessed 1 week after stopping the cuprizone diet. Similar to the observations made during early remyelination, aSyn deficiency did not affect remyelination pattern and numbers of CNS cells after 7 days of remyelination (Fig. 6a–h, Appendix). Matching these data, performance on the Rotarod did not significantly differ between aSyn^+/+^ and aSyn^−/−^ mice at this time point (Fig. 6i, Appendix).

## Discussion

aSyn is known to play an important role in the pathology of various neurodegenerative and neuroinflammatory disorders. Here we analyzed the impact of aSyn deficiency at the late stage of EAE, 8 weeks after immunization with MOG_35–55_ peptide. A comprehensive histological analysis of spinal cord sections revealed decreased numbers of CNS infiltrating T cells and mononuclear phagocytes in late-stage EAE lesions of aSyn^−/−^ mice, accompanied by reduced axonal and myelin loss as compared with endogenous aSyn-expressing littermate controls (aSyn^+/+^). In a previous study, we investigated the effect of aSyn deficiency in the onset phase of EAE and demonstrated that aSyn is involved in the regulation of immunological responses. T helper 1 cells of aSyn^−/−^ mice showed a hyperproliferative phenotype which was associated with increased IL-2 expression and increased infiltration of T cells into the spinal cord (Ettle et al. 2016b). In line with these data, aSyn deficiency has been reported to promote a hyperproliferative phenotype and to affect T cell development (Shameli et al. 2016). Since, in the EAE model, demyelination and axonal loss is triggered by the invasion of autoreactive T cells and other peripheral immune cells into the CNS, the present data further indicate that aSyn deficiency modulates peripheral immune responses thereby affecting demyelinating processes (Constantinescu et al. 2011). Whether the reduction of myelin loss in the phase of late-stage demyelination is due to T cell exhaustion following hyperproliferation observed in the early stages of EAE or caused by enhanced clearance of myelin debris and apoptotic cells in aSyn^−/−^ mice still remains to be elucidated. Previously, it was shown that increased levels of aSyn reduce phagocytic activity of microglial cells and macrophages in an animal model of PD as well as in human cells (Gardai et al. 2013). In MSA and its corresponding mouse models, increased levels of oligodendroglial aSyn are accompanied by astro- and microgliosis already indicating that aSyn may play an important role during neuroinflammation in synucleinopathies (Stefanova et al. 2005; Ahmed et al. 2012). Overall, results from both early and late phase EAE studies support the notion that aSyn is involved in the regulation of central and peripheral inflammatory processes.

In order to provide further evidence for a role in regulating demyelinating processes in late-stage EAE, future studies investigating the impact of aSyn deficiency on the peripheral versus central immune system in late-stage inflammation are required. Analogous to previous analysis in the early EAE phase, flow cytometry analysis of splenocytes or adoptive transfer EAE experiments using T cells isolated from aSyn-deficient mice may help to elucidate the role of aSyn during immune responses in more detail (Ettle et al. 2016b). Another possibility to more closely investigate underlying mechanisms is to induce active EAE in conditional knock-out mice specifically lacking aSyn in the central nervous system or in the peripheral immune compartment.

In the present study, evidence for an immune-mediated mechanism of demyelination also comes from the results obtained by cuprizone-induced toxic de- and remyelination. In this model, demyelination is induced by feeding mice with the chopper chelator cuprizone which selectively depletes mature oligodendrocytes (Bénardais et al. 2013). While this treatment leads to a neuroinflammatory response in the CNS characterized by micro- and astrogliosis, peripheral immune cells play a minor role (Hiremath et al. 1998; Torkildsen et al. 2009). Using this approach, we did not detect striking differences between aSyn^+/+^ and aSyn^−/−^ mice regarding de- and remyelinating and neuroinflammatory processes. Surprisingly, aSyn deficiency showed no direct impact on oligodendroglial cells. Based on previous findings indicating that aSyn overexpression affects OPC maturation in vitro and in vivo*,* we originally hypothesized that lack of aSyn might also show effects on oligodendrocyte lineage cells (Ettle et al. 2014, 2016a; May et al. 2014). However, the models used for the present experiments might account for these observations. In the EAE model, demyelination and oligodendrocyte injury are triggered by strong immunological responses. Considering that aSyn has also been described as modulator of inflammatory processes, these effects most probably conceal CNS-endogenous detrimental events. This makes it difficult to dissect potential primary effects of aSyn deficiency on oligodendroglial cells and demyelination from immune-mediated effects (Gardai et al. 2013; Shameli et al. 2016). Accordingly, the observed increase in OPCs in lesions of aSyn^+/+^ mice might be rather due to increased recruitment of OPCs triggered by pronounced inflammation and tissue damage in these animals. Furthermore, cuprizone is a very strong toxin selectively affecting mature oligodendrocytes, but the exact mechanism has not been completely understood yet. Therefore, in this demyelination model, effects of aSyn deficiency on de- and remyelination processes might be overwhelmed by these strong toxic effects.

Despite strenuous research efforts, effective strategies to prevent CNS demyelination or to induce remyelination are still missing and the exact mechanisms underlying autoimmune myelin destruction are incompletely understood. This emphasizes the importance to unravel factors triggering demyelination. Expression of oligodendroglial aSyn has been shown to be an important feature in the neuropathology of MSA inducing a regional-specific pattern not only of demyelination but also of severe inflammation (Ishizawa et al. 2004; Ahmed et al. 2012; Ettle et al. 2016a; Hoffmann et al. 2019). A combined therapy of anti-inflammatory targets and immunotherapy against aSyn has shown promising results in preclinical studies of MSA (Valera et al. 2017). Whether this therapeutic strategy might also show beneficial results in early- and late-stage EAE needs to be investigated in the future.

Of note, a recent study implicated beta-Synuclein (bSyn), a member of the synuclein family with strong homology to aSyn, in T cell–mediated pathology. Enhanced numbers of bSyn-reactive T cells have been detected in the blood of chronic progressive MS patients. Moreover, it has been shown that bSyn-specific T cells were highly pathogenic as adoptive transfer elicited heterogeneous EAE symptoms in Lewis rats and specifically induced severe inflammation in the gray matter of the brain ultimately resulting in permanent damage (Lodygin et al. 2019). Lately, pronounced numbers of aSyn-reactive T cells have been detected in PD patients, and strikingly also in individuals suffering from MS (Sulzer et al. 2017; Lodygin et al. 2019). Considering that in our study aSyn deficiency partly protected from late-stage demyelination, it will be of particular interest to determine whether aSyn may also serve as target of CNS autoimmune reactions. Importantly, the identification of CNS target antigens is imperative for the development of highly selective immunotherapies preventing demyelination caused by autoreactive processes.

In summary, the data presented in this study so far indicate that aSyn acts as modulator of peripheral autoimmune-mediated neuroinflammatory demyelination. This further identifies aSyn as a regulator of immune responses and renders this protein a potential bridging element between the peripheral immune system and the CNS in demyelinating diseases including MS.
